# Association between asthma or chronic obstructive pulmonary disease and chronic otitis media

**DOI:** 10.1038/s41598-022-08287-w

**Published:** 2022-03-10

**Authors:** Sung Kyun Kim, Seok Jin Hong, Dae Myoung Yoo, Chanyang Min, Hyo Geun Choi

**Affiliations:** 1 Department of Otorhinolaryngology-Head and Neck Surgery, Hallym University College of Medicine, Dongtan, Korea; 2 Laboratory of Brain & Cognitive Sciences for Convergence Medicine, Hallym University College of Medicine, Anyang, Korea; 3 Hallym Data Science Laboratory, Hallym University College of Medicine, Anyang, Korea; 4 Graduate School of Public Health, Seoul National University, Seoul, Korea; 5 Department of Otorhinolaryngology-Head and Neck Surgery, Hallym University College of Medicine, Hallym University Sacred Heart Hospital, 22, Gwanpyeong-ro 170, Anyang, Gyeonggi 14068 Republic of Korea

**Keywords:** Epidemiology, Asthma, Chronic obstructive pulmonary disease

## Abstract

We hypothesized that asthma/chronic obstructive pulmonary disease (COPD) might increase the risk of chronic otitis media (COM), as asthma or COPD affects other diseases. The aim of this research was to investigate whether the incidence of COM is affected by a diagnosis of asthma or COPD in patients compared to matched controls from the national health screening cohort. A COM group (n = 11,587) and a control group that was 1:4 matched for age, sex, income, and residence area (n = 46,348) were selected. The control group included participants who never received treatment for COM from Korean National Health Insurance Service-Health Screening Cohort from 2002 to 2015. The crude and adjusted odds ratios (ORs) of previous asthma/COPD before the index date for COM were analyzed using conditional logistic regression. The analyses were stratified by age, sex, income, and region of residence. The period prevalence of asthma (17.5% vs. 14.3%, p < 0.001) and COPD (6.6% vs. 5.0%, p < 0.001) were significantly higher in the COM group than in the control group. In addition, the odds of asthma and COPD were significantly higher in the COM group than in the control group. Both asthma (adjusted OR 1.23, 95% confidence interval [CI] 1.16–1.31, p < 0.001) and COPD (adjusted OR 1.23, 95% CI 1.13–1.35, p < 0.001) increased the ORs for COM. This positive association between asthma/COPD and COM indicates that asthma/COPD might increase the incidence of COM.

## Introduction

Asthma and chronic obstructive pulmonary disease (COPD) are two of the most prevalent pulmonary diseases, and people with these conditions may experience various symptoms related to airflow limitation^1^. Asthma is a common inflammatory disease of the lower airway and is characterized by airway obstruction and hyperresponsiveness^2^. Various comorbidities, such as chronic sinusitis, rhinitis, gastroesophageal reflux disease (GERD), anxiety and depression, that are related to asthma via crosstalk among numerous cellular and humoral mediators have recently been reported through analysis of large databases^3–6^. COPD is characterized by a persistent limitation of airflow due to an increase in forced vital capacity and/or a decrease in forced expiratory volume, and it is the fourth leading cause of death^1^. The comorbid conditions associated with COPD have been widely reported and can influence the clinical course and management of COPD^7^. Several published papers have indicated that COPD is associated with an increased incidence of lung cancer, pulmonary fibrosis, hypertension, congestive heart failure, coronary heart disease, diabetes, osteoporosis, chronic mental illnesses, obstructive sleep apnea, and GERD^8–10^.

Chronic otitis media (COM) can be defined as persistent inflammation occurring in the middle ear and mastoid cavity and is one of the most prevalent diseases among otologic diseases. COM causes hearing loss, which can lead to cognitive dysfunction such as dementia and Alzheimer's disease as well as social deprivation^11,12^. Dysfunction of the eustachian tube (ETD) and recurrent microbial infections obstruct the ventilation route, resulting in inflammatory lesions such as granulation and tympanosclerotic plaque in the middle ear cavity. In addition, the complex interaction of immune responses and biochemical factors contributes to the recurrence and chronicity of the disease^13,14^. The prevalence of COM varies among countries and is higher in developing countries than in other countries^12^. The weighted prevalence of COM in South Korea was 3.8% according to the Korean National Health and Nutrition Examination Survey in 2012^15^. Factors related to the prevalence of COM are a history of otitis media in childhood, access to medical facilities, antibiotic use, exposure to pollutants, poor hygiene, and direct/secondhand smoke, in addition to poor ventilation due to ETD.

In the respiratory tract of asthma and COPD patients, tissue remodeling occurs due to airflow limitation, hypoxia, and chronic inflammation, which affects interactions of the microbial community in the respiratory mucosa, including the Eustachian tube and middle ear^16,17^. Alteration of the microbial community in chronic respiratory diseases such as asthma and COPD leads to a decrease in mucociliary clearance and oxygen availability of adjacent upper respiratory mucosa. This is known to affect the occurrence of chronic rhinosinusitis, chronic inflammation of the nasal cavity and paranasal sinus, and otitis media in children in which pathogens are introduced into the middle ear through the Eustachian tube^18–20^.

However, most of the studies on the association between asthma or COPD and otitis media have focused on eosinophilic otitis media or chronic suppurative otitis with effusion in children rather than on COM in adults. Here, this study aimed to demonstrate whether asthma and COPD can be independent risk factors for COM using data obtained from a national health screening cohort.

## Results

The period prevalence of asthma was 17.5%, and that of COPD was 6.6% in the COM group during the follow-up period, which was significantly higher than that in the control group (*p* < 0.001). The distributions of age group, sex, income level, and type of residence region were comparable between the COM and control groups. The systolic blood pressure and diastolic blood pressure of the COM group were significantly lower than those of the control group. However, there was no significant difference in cholesterol level, fasting glucose level, or the degree of obesity classified according to BMI between the two groups. The numbers of past smokers (10.4%) and nonsmokers (74.6%) in the COM group were significantly higher, and the number of current smokers (15.0%) was significantly lower than those in the control group (16.4%). Moreover, the frequency of alcohol consumption was significantly lower in the COM group (27.3% in ≥ 1 time a week) than in the control group (28.9% in ≥ 1 time a week). CCI scores related to comorbidities were calculated excluding pulmonary diseases, and the number of participants with more than 1 point was mainly from the COM group (31.8% vs 29.4% in control group) (Table 1).

**Table 1:** General characteristics of participants.

Characteristics	Total participants		
	COM	Control	p-value
**Age (years old, n, %)**			1.000
40–44	315 (2.7)	1260 (2.7)	
45–49	1318 (11.4)	5272 (11.4)	
50–54	1962 (16.9)	7848 (16.9)	
55–59	2088 (18.0)	8352 (18.0)	
60–64	1887 (16.3)	7548 (16.3)	
65–69	1729 (14.9)	6916 (14.9)	
70–74	1273 (11.0)	5092 (11.0)	
75–79	669 (5.8)	2676 (5.8)	
80–84	282 (2.4)	1128 (2.4)	
85 +	64 (0.6)	256 (0.6)	
**Sex (n, %)**			1.000
Males	5430 (46.9)	21,720 (46.9)	
Females	6157 (53.1)	24,628 (53.1)	
**Income (n, %)**			1.000
1 (lowest)	1889 (16.3)	7556 (16.3)	
2	1566 (13.5)	6264 (13.5)	
3	1896 (16.4)	7584 (16.4)	
4	2530 (21.8)	10,120 (21.8)	
5 (highest)	3706 (32.0)	14,824 (32.0)	
**Region of residence (n, %)**			1.000
Urban	4824 (41.6)	19,296 (41.6)	
Rural	6763 (58.4)	27,052 (58.4)	
Total cholesterol (mg/dL, mean, SD)	199.0 (38.1)	199.7 (38.2)	0.080
SBP (mmHg, mean, SD)	126.5 (16.9)	127.1 (17.2)	0.001†
DBP (mmHg, mean, SD)	78.2 (10.7)	78.6 (10.9)	0.001†
Fasting blood glucose (mg/dL, mean, SD)	99.6 (29.9)	100.2 (29.4)	0.103
**Obesity (n, %)**^‡^			0.083
Underweight	255 (2.2)	1180 (2.6)	
Normal	4049 (34.9)	16,340 (35.3)	
Overweight	3244 (28.0)	12,617 (27.2)	
Obese I	3700 (31.9)	14,752 (31.8)	
Obese II	339 (2.9)	1459 (3.2)	
**Smoking status (n, %)**			0.002*
Nonsmoker	8641 (74.6)	34,055 (73.5)	
Past smoker	1204 (10.4)	4707 (10.2)	
Current smoker	1742 (15.0)	7586 (16.4)	
**Alcohol consumption (n, %)**			0.001*
< 1 time a week	8422 (72.7)	32,953 (71.1)	
≥ 1 time a week	3165 (27.3)	13,395 (28.9)	
**CCI score (score, n, %)**^§^			< 0.001*
0	7897 (68.2)	32,698 (70.6)	
1	1726 (14.9)	5989 (12.9)	
2	901 (7.8)	3629 (7.8)	
3	488 (4.2)	1742 (3.8)	
≥ 4	575 (5.0)	2290 (4.9)	
COPD (n, %)	763 (6.6)	2317 (5.0)	< 0.001*
Asthma (n, %)	2028 (17.5)	6610 (14.3)	< 0.001*

*CCI* Charlson comorbidity index, *COM* chronic otitis media, *COPD* chronic obstructive pulmonary disease, *DBP* diastolic blood pressure, *SBP* systolic blood pressure, *SD* standard deviation.

*Chi-square test. Significance at p < 0.05.

^†^Wilcoxon rank-sum test. Significance at p < 0.05.

^‡^Obesity (BMI, body mass index, kg/m^2^) was categorized as < 18.5 (underweight), ≥ 18.5 to < 23 (normal), ≥ 23 to < 25 (overweight), ≥ 25 to < 30 (obese I), and ≥ 30 (obese II).

^§^CCI scores were calculated without pulmonary disease.

The adjusted OR of asthma for COM was 1.23 (95% CI 1.16–1.31, *p* < 0.001), which was significantly higher regardless of age group, sex, income level, and residential area. In addition, the adjusted ORs of asthma were higher in the younger-than-60-years-old, female, low-income, and urban-living groups (Table 2). The adjusted OR of COPD for COM was 1.23 (95% CI 1.13–1.35, *p* < 0.001), which was significantly higher for all ages, both sexes, all income levels, and both regions of residence, as observed for asthma. Moreover, the adjusted ORs of COPD were higher in participants aged 60 and over, women, those with a high income, and those living in rural areas (Table 3).

**Table 2:** Odds ratios (95% confidence interval) of asthma for chronic otitis media with subgroup analyses according to age, sex, income, and region of residence.

Characteristics	No. of COM/No. of participants (%)	Odds ratios for COM			
		Crude^†^	p-value	Adjusted^†,‡^	p-value
**Total participants (n = 57,935)**					
Asthma	2028/8638 (23.5)	1.29 (1.22–1.36)	< 0.001*	1.23 (1.16–1.31)	< 0.001*
Control	9559/49,297 (19.4)	1		1	
**Age < 60 years old (n = 28,415)**					
Asthma	717/2899 (24.7)	1.37 (1.25–1.50)	< 0.001*	1.34 (1.22–1.47)	< 0.001*
Control	4966/25,516 (19.5)	1		1	
**Age ≥ 60 years old (n = 29,520)**					
Asthma	1311/5739 (22.8)	1.24 (1.16–1.33)	< 0.001*	1.18 (1.09–1.27)	< 0.001*
Control	4593/23,781 (19.3)	1		1	
**Males (n = 27,150)**					
Asthma	771/3302 (23.4)	1.27 (1.16–1.38)	< 0.001*	1.20 (1.09–1.32)	< 0.001*
Control	4659/23,848 (19.5)	1		1	
**Females (n = 30,785)**					
Asthma	1257/5336 (23.6)	1.30 (1.21–1.40)	< 0.001*	1.26 (1.17–1.36)	< 0.001*
Control	4900/25,449 (19.3)	1		1	
**Low income (n = 26,755)**					
Asthma	978/4111 (23.8)	1.32 (1.22–1.43)	< 0.001*	1.27 (1.17–1.38)	< 0.001*
Control	4373/22,644 (19.3)	1		1	
**High income (n = 31,180)**					
Asthma	1050/4527 (23.2)	1.26 (1.17–1.36)	< 0.001*	1.20 (1.11–1.30)	< 0.001*
Control	5186/26,653 (19.5)	1		1	
**Urban (n = 24,120)**					
Asthma	825/3369 (24.5)	1.37 (1.26–1.50)	< 0.001*	1.33 (1.21–1.45)	< 0.001*
Control	3999/20,751 (19.3)	1		1	
**Rural (n = 33,815)**					
Asthma	1203/5269 (22.8)	1.23 (1.15–1.32)	< 0.001*	1.17 (1.09–1.27)	< 0.001*
Control	5560/28,546 (19.5)	1		1	

*CCI* Charlson comorbidity index, *COM* chronic otitis media, *COPD* chronic obstructive pulmonary disease, *DBP* diastolic blood pressure, *SBP* systolic blood pressure.

*Conditional logistic regression, Significance at p < 0.05.

^†^Models were stratified by age, sex, income, and region of residence.

^‡^Adjusted for obesity, smoking, alcohol consumption, CCI scores, total cholesterol, SBP, DBP, fasting blood glucose, and COPD.

**Table 3:** Odds ratios (95% confidence interval) of COPD for COM with subgroup analyses according to age, sex, income, and region of residence.

Characteristics	No. of COM/No. of participants (%)	Odds ratios for COM			
		Crude^†^	p-value	Adjusted^†,‡^	p-value
**Total participants (n = 57,935)**					
COPD	763/3080 (24.8)	1.36 (1.25–1.48)	< 0.001*	1.23 (1.13–1.35)	< 0.001*
Control	10,824/54,855 (19.7)	1		1	
**Age < 60 years old (n = 28,415)**					
COPD	151/591 (25.5)	1.38 (1.15–1.67)	0.001*	1.24 (1.02–1.50)	0.031*
Control	5532/27,824 (19.9)	1		1	
**Age ≥ 60 years old (n = 29,520)**					
COPD	612/2489 (24.6)	1.35 (1.23–1.49)	< 0.001*	1.25 (1.13–1.39)	< 0.001*
Control	5292/27,031 (19.6)	1		1	
**Males (n = 27,150)**					
COPD	394/1632 (24.1)	1.32 (1.17–1.49)	< 0.001*	1.20 (1.06–1.37)	0.005*
Control	5036/25,518 (19.7)	1		1	
**Females (n = 30,785)**					
COPD	369/1448 (25.5)	1.40 (1.24–1.59)	< 0.001*	1.27 (1.12–1.44)	< 0.001*
Control	5788/29,337 (19.7)	1		1	
**Low income (n = 26,755)**					
COPD	372/1505 (24.7)	1.36 (1.20–1.53)	< 0.001*	1.21 (1.06–1.38)	0.005*
Control	4979/25,250 (19.7)	1		1	
**High income (n = 31,180)**					
COPD	391/1575 (24.8)	1.36 (1.21–1.54)	< 0.001*	1.25 (1.10–1.42)	0.001*
Control	5845/29,605 (19.7)	1		1	
**Urban (n = 24,120)**					
COPD	258/1024 (25.2)	1.38 (1.19–1.60)	< 0.001*	1.23 (1.06–1.43)	0.008*
Control	4566/23,096 (19.8)	1		1	
**Rural (n = 33,815)**					
COPD	505/2056 (24.6)	1.35 (1.21–1.50)	< 0.001*	1.24 (1.11–1.39)	< 0.001*
Control	6258/31,759 (19.7)	1		1	

*CCI* Charlson comorbidity index, *COM* chronic otitis media, *COPD* chronic obstructive pulmonary disease, *DBP* diastolic blood pressure, *SBP* systolic blood pressure.

*Conditional logistic regression, Significance at p < 0.05.

^†^Models were stratified by age, sex, income, and region of residence.

^‡^Adjusted for obesity, smoking, alcohol consumption, CCI scores, total cholesterol, SBP, DBP, fasting blood glucose, and asthma.

The ORs of asthma with COM in model 2 adjusted for age group, sex, income, region of residence, obesity, smoking, alcohol consumption, CCI scores, total cholesterol, SBP, DBP, fasting blood glucose, and asthma were significantly higher in all subgroups except the group with CCI score 1 (Supplementary Table S1). Additionally, the association of COPD with COM in the same model as Supplementary Table S1 was consistent in all subgroups except the BMI < 18.5, BMI ≥ 23 to < 25, and borderline cholesterol level (≥ 200 to < 240 mg/dL) groups (Supplementary Table S2).

## Discussion

The ORs for asthma and COPD were significantly higher in the COM group than in the control group. Additionally, the ORs of asthma and COPD in the COM group were considerably higher than those in the control group in all age, both sex, all income level, and both residential area groups.

Previous studies on the association between asthma and otitis media were conducted in children^21,22^. Otitis media (OR 1.8; 95% CI 1.2–2.6) in the first year was related to the presence of asthma at 4 years of age in the Oslo Birth Cohort^21^. In the nationwide population cohort of Korea, asthma and otitis media showed a reciprocal association (hazard ratio [HR] 1.46; 95% CI 1.40–1.52, *p* < 0.001 for otitis media, HR 1.43; 95% CI 1.36–1.50, *p* < 0.001 for asthma) in children^22^. However, these studies postulated a relationship between otitis media and asthma in children, unlike our study on COM in adults, and no studies on the epidemiological association between the two diseases have been demonstrated in adults until now. Since asthma patients have increased airway hypersensitivity, the course of the disease is affected by several primary or secondary factors, which can be associated with COM. In particular, exposure to urban fine/ultrafine particles that have a large surface area, are easily deposited in the airways and can inhibit phagocytosis, affects not only the airway but also the eustachian tube and middle ear^23^. Urban particles cause mucosal thickening and inflammatory cell infiltration in the middle ear due to epithelial cells and vascular space widening and play a role in decreasing ENaC-α expression and increasing the level of MUC5AC expression^24^. In addition, factors such as respiratory infection and allergen exposure affect ventilation and mucociliary clearance of the eustachian tube and/or middle ear, which is thought to be able to potentiate the onset of COM^25,26^.

To the best of our knowledge, no studies have reported the epidemiological association between COPD and otitis media. However, several causative factors of COPD can directly or indirectly affect the eustachian tube and middle ear epithelium. Smoking and tobacco use were major determinants of COPD and had a significantly higher odds ratio for overall middle ear diseases (adjusted OR 1.15; 95% CI 0.99–1.33, *p* = 0.05), and the adjusted OR in men aged 40–60 years was 1.73 (95% CI 1.29–2.30, *p* < 0.001), which was particularly high compared to other age groups^27^. Exposure to cigarette smoke solution in human middle ear epithelial cells upregulated TNF-α, EGFR and MUC5AC mRNA levels and caused histological changes such as cilia loss, prominent squamous metaplasia, and goblet cell depletion in the eustachian tube^28,29^. Additionally, the association between COPD and COM can be elucidated through several bacterial pathogens that are commonly involved in both diseases. *Nontypeable Haemophilus influenzae* and *Moraxella catarrhalis* are representative bacterial species that contribute to COPD exacerbation in smokers, and acute and recurrent otitis media are prevalent in young children^30,31^. *Streptococcus pneumoniae*, which colonizes the human nasopharynx, also causes airway inflammation, compromises the mucociliary system of the airway epithelium, and promotes bacterial overgrowth in chronic respiratory diseases such as allergic asthma and COPD^32,33^. In addition, recurrent infection and persistent inflammation in chronic respiratory diseases such as COPD and asthma change the composition of the microbial community not only in the adjacent airway, nasopharynx, and paranasal sinuses but also in the eustachian tube and middle ear epithelium^34,35^.

Mucus plugging of the sterile airway in asthma and COPD causes hypoxia and necrosis of airway epithelial cells, leading to neutrophilic airway inflammation^36^. Interleukin 1 receptor (IL-1R) plays a key role in activating neutrophilic inflammation and airway remodeling in the mucus-abundant airway^37^ and impairing antibacterial host defense mechanisms through Toll-like receptors (TLRs) and extracellular signal-regulated kinase (ERK) signaling pathways^38–40^. This airway inflammation can promote swelling and narrowing of the eustachian tube mucosa, compromise ventilation and mucociliary clearance, and eventually accelerate the accumulation of pathogens in the middle ear cavity in chronic cases^41^.

This study demonstrates epidemiological associations between asthma/COPD and COM known so far using nested case–control study design from a large, nationwide population database. Additionally, expected confounding factors such as age group, sex, income level, and area of residence were matched in the control group at a 1:4 ratio to enhance the reliability of our results. The health check-up database by life cycle for the national population contains not only demographic data but also objective data such as blood pressure, fasting glucose level, and quantified information related to individual habits. Thus, it has the advantage of providing additional information about the association between diseases primarily based on medical claim codes and prescribed medication. Although this study postulates that asthma/COPD increases the occurrence of COM, we have several limitations. First, the causality between asthma/COPD and COM could not be sufficiently concluded due to the retrospective research design. Second, it is difficult to obtain descriptive information related to asthma, COPD, and COM, such as microbiological culture results, hearing threshold, radiological findings, and pulmonary function test results, from the data included in the health screening cohort database. Although several confounding factors were matched to increase the statistical power of association between diseases, the absence of specific information such as severity and clinical progression of each disease did not completely exclude the possibility of potential misdiagnosis. Also, unlike COPD, asthma is a disease that can start from childhood, but our dataset cannot obtain information on the childhood histories of patients diagnosed with asthma during the follow-up period. This study includes wide range of otitis media from suppurative through non-suppurative otitis media. The difference of these pathogenesis might affect the results. Last, the number of medical encounters in patients with COM may increase compared with that in the control group due to the chronic clinical aspect of COM, but we did not correct for this. Therefore, this could be a possible confounder that may have influenced the timing of diagnosis of asthma and COPD. In future studies, a data analysis that can take into account the number of medical encounters should be devised to minimize confounding bias that affects the incidence of diseases.

## Materials and methods

### Study population

The ethics committee of Hallym University (2019-10-023) approved this study. The requirement for written informed consent was waived by the Institutional Review Board of Hallym University. All analyses adhered to the guidelines and regulations of the ethics committee of Hallym University. The data was used from the Korean National Health Insurance Service-Health Screening Cohort (NHIS-HEALS). A detailed description of the NHIS-HEALS data has been published previously^42^. The Korean National Health Insurance Service (NHIS) randomly chooses about 10% of nationwide population who underwent health check-up from 2002 through 2015^43^. Age and gender-specific distributions of population in cohort are described online (http://nhiss.nhis.or.kr). All Koreans over the age of 40 with a 13-digit resident registration number are requested to have a health evaluation biannually without cost. All medical records and treatments, as well as births and deaths, are managed under the Korean Health Insurance Review and Assessment system based on a 13-digit resident registration number.

This cohort database from NHIS includes (i) personal information, (ii) medical claim code related to procedures and prescriptions, (iii) diagnostic code using the International Classification of Disease-10 (ICD-10), (iv) socioeconomic data (residence and income), (v) death records from the Korean National Statistical Office (vi) health examination data (height, weight, drinking, smoking habit, blood pressure, urinalysis, hemoglobin, fasting glucose, lipid parameters, creatinine, and liver enzymes) for each participant over the period from 2002 to 2015.

### Participant selection

The COM group was selected from among 514,866 participants with 615,488,428 medical claim codes (n = 16,594). The control group was chosen from all participants who were not involved in the COM group during 2002–2015 (n = 498,272). To select COM participants with a first-time diagnosis, we excluded COM participants from 2002 through 2003 for washout (n = 4974). For the control participants, we excluded 1,516 participants who died before 2004 or had no records since 2004. Control participants who had the ICD-10 codes H65.2 (chronic serous otitis media), H65.3 (chronic mucoid otitis media), H65.4 (other chronic nonsuppurative otitis media), H65.9 (nonsuppurative otitis media, unspecified), H66.1 (chronic tubotympanic suppurative otitis media), H66.2 (chronic atticoantral suppurative otitis media), H66.3 (other chronic suppurative otitis media), or H66.4 (suppurative otitis media, unspecified) once between 2002 and 2015 were excluded (n = 44,843). Participants who were diagnosed ≥ 2 times with malignant neoplasms of the meninges (C70), malignant neoplasms of the brain (C71), or malignant neoplasms of the spinal cord, cranial nerves and other parts of the central nervous system (C72) were excluded from the COM group (n = 32) and the control group (n = 798). A participant who did not have blood pressure records was excluded from the COM group (n = 1).

**Figure 1:**
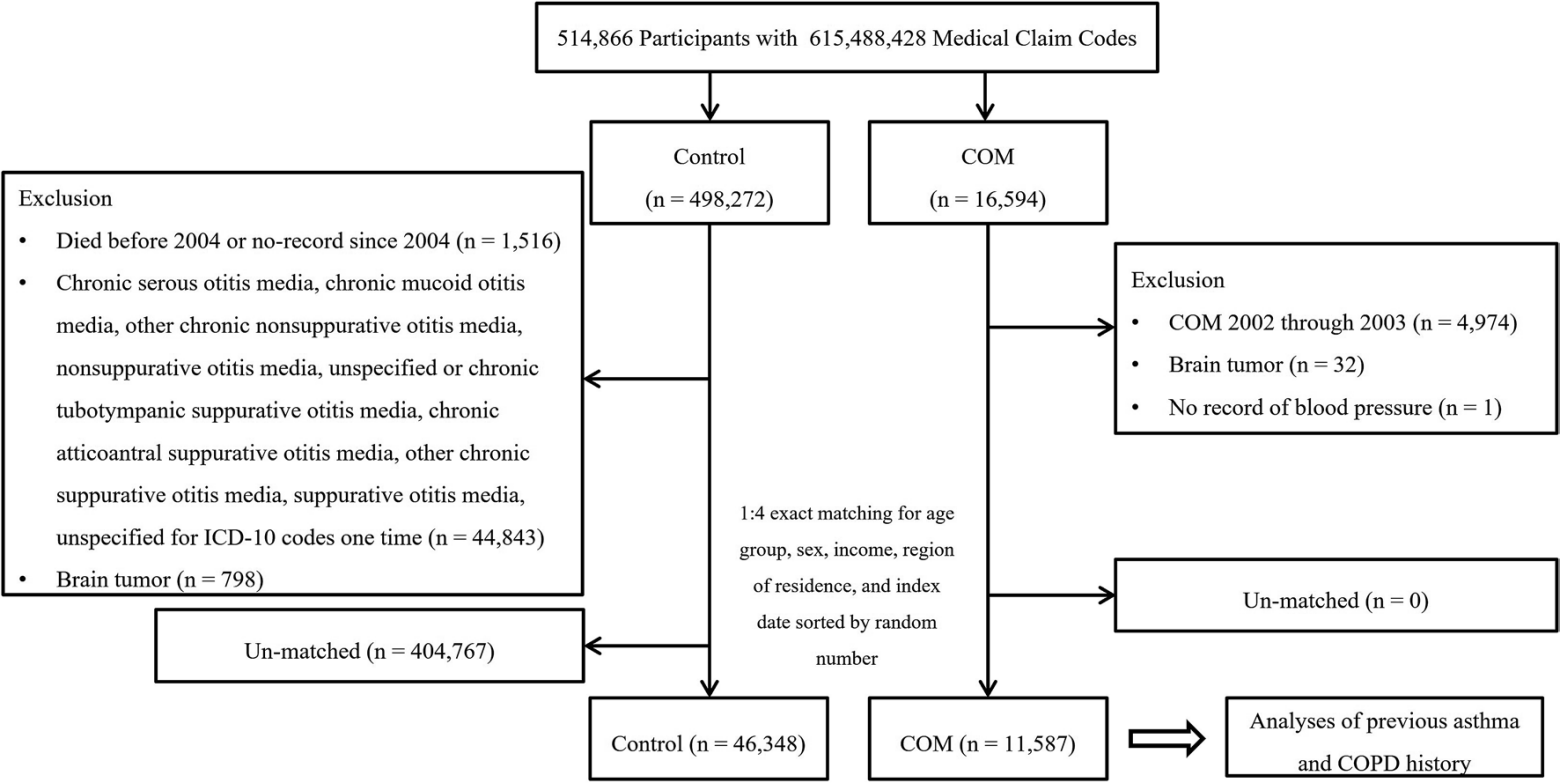
A schematic illustration of the participant selection process. Of a total of 514,866 participants, 11,587 of COM participants were matched with 46,348 of control participants for age, sex, income, and region of residence.

COM participants were 1:4 matched with control participants for age group, sex, income, and region of residence. To minimize selection bias, the control participants were sorted by random number order. The index date of each COM participant was set as the time of diagnosis of COM. The index dates of the control participants were set as the index date of their matched COM participants. Therefore, each matched COM participant had the same index date as their control participants. During the matching process, 404,767 control participants were excluded. Cases with brain tumors and cases without medical records were excluded in both groups. Also, deaths before 2004 were excluded because they did not belong to any group. Cases (n = 4974) diagnosed with COM between 2002 and 2003 that did not meet the definition of index date were excluded in the control group. In addition, we considered excluding the ICD-10 code for COM from the control group is to completely exclude patients with COM included in the control group. Finally, 11,587 COM participants were 1:4 matched with 46,348 control participants for the study (Fig. 1).

### Independent variables (asthma and chronic obstructive pulmonary disease)

Asthma was defined if participants were treated for asthma (ICD-10: J45) or status asthmaticus (J46) from 2002 through 2015 ≥ 2 times with asthma-related medications prescribed ≥ 2 times, as in our previous study^44^. This method was modified from a previously validated study^45^.

Chronic obstructive pulmonary disease (COPD) was defined by unspecified chronic bronchitis (J42), emphysema (J43), or other COPD (J44) except MacLeod syndrome (J430) ≥ 2 times with COPD-related medications prescribed ≥ 2 times following a previous study^46^.

### Dependent variable (chronic otitis media)

COM was defined if the participants were diagnosed with the ICD-10 codes H65.2 (chronic serous otitis media), H65.3 (chronic mucoid otitis media), H65.4 (other chronic nonsuppurative otitis media), H65.9 (nonsuppurative otitis media, unspecified), H66.1 (chronic tubotympanic suppurative otitis media), H66.2 (chronic atticoantral suppurative otitis media), H66.3 (other chronic suppurative otitis media), or H66.4 (suppurative otitis media, unspecified) by a physician ≥ 2 times.

### Covariates

Participants were divided into age groups by 5-year intervals: 40–44, 45–49, 50–54…, and 85+ years old. A total of 10 age groups were specified. In addition, participants were classified into 5 income groups (class 1 [lowest income]–5 [highest income]). The region of residence was classified as urban and rural areas, following our previous study^42^. Metabolic factors reported to be associated with the incidence of COM, COPD, and asthma such as tobacco smoking, alcohol consumption, obesity (using BMI), total cholesterol (mg/dL), systolic blood pressure (SBP, mmHg), diastolic blood pressure (DBP, mmHg), and fasting blood glucose (mg/dL) were used as described in our previous study^15,42,47–51^. The Charlson Comorbidity Index (CCI) was calculated to measure the burden of comorbidities except for pulmonary disease from 0 through 29 scores^52^.

### Statistical analyses

The general characteristics of the COM and control groups were compared using the McNemar's chi-square test for categorical variables and the Wilcoxon sign-rank test for continuous variables.

To analyze the odds ratios (ORs) with 95% confidence intervals (CIs) of asthma or COPD for COM, conditional logistic regression was used. In these analyses, crude and adjusted models were calculated. For asthma as the independent variable, the adjusted model was adjusted for obesity, smoking, alcohol consumption, CCI scores, total cholesterol, SBP, DBP, fasting blood glucose, and COPD. For COPD as the independent variable, asthma was used as the covariate instead of COPD in the adjusted model. The analyses were stratified by age, sex, income, and region of residence.

For the subgroup analyses, we divided participants by age (< 60 years old and ≥ 60 years old), sex (males and females), income (low income and high income), and region of residence (urban and rural). We analyzed crude and adjusted models using conditional logistic regression.

Additionally, we performed subgroup analyses according to obesity, smoking, alcohol consumption, total cholesterol, blood pressure, fasting blood glucose, and CCI using unconditional logistic regression (Supplementary Tables S1 and S2). Model 1 (adjusted for age, sex, income, and region of residence) and model 2 (model 1 plus obesity, smoking, alcohol consumption, CCI scores, total cholesterol, SBP, DBP, fasting blood glucose, and COPD [or asthma]) were calculated for additional subgroup analyses.

For the statistical analyses, SAS version 9.4 (SAS Institute Inc., Cary, NC, USA) was used. We performed two-tailed analyses, and significance was defined as p values less than 0.05.

## Supplementary Information

Supplementary Table S1.

Supplementary Table S2.
