# Supplementary material for: Association between asthma or chronic obstructive pulmonary disease and chronic otitis media
Source: Sci Rep. 2022 Mar 10;12:4228. doi: 10.1038/s41598-022-08287-w (PMC8913729; doi:10.1038/s41598-022-08287-w)
Supplement: Supplementary file 1 — Supplementary Table S1. [file 41598_2022_8287_MOESM1_ESM.docx]

**S1 Table** Subgroup analyses of odds ratios (95% confidence interval) of asthma for COM according to obesity, smoking, alcohol consumption, total cholesterol, blood pressure, blood glucose, and CCI score

| Characteristics | | | No. of COM/ No. of participants (%) | | Odds ratios for COM | | | |
| --- | --- | --- | --- | --- | --- | --- | --- | --- |
|  |  |  |  |  | Model 1† | P-value | Model 2‡ | P-value |
| Obesity | | | | | | | | |
|  | Underweight (n = 1,435) | | | | | | | |
|  |  | Asthma | 58/240 (24.2) | | 1.57 (1.12-2.21) | 0.010* | 1.66 (1.14-2.43) | 0.008* |
|  |  | Control | 197/1,195 (16.5) | | 1 |  | 1 |  |
|  | Normal weight (n = 20,389) | | | | | | | |
|  |  | Asthma | 638/2,746 (23.2) | | 1.27 (1.15-1.40) | <0.001* | 1.20 (1.08-1.33) | 0.001* |
|  |  | Control | 3,411/17,643 (19.3) | | 1 |  | 1 |  |
|  | Overweight (n = 15,861) | | | | | | | |
|  |  | Asthma | 541/2,241 (24.1) | | 1.29 (1.16-1.44) | <0.001* | 1.26 (1.13-1.41) | <0.001* |
|  |  | Control | 2,703/13,620 (19.8) | | 1 |  | 1 |  |
|  | Obese (n = 20,250) | | | | | | | |
|  |  | Asthma | 791/3,411 (23.2) | | 1.28 (1.17-1.40) | <0.001* | 1.22 (1.11-1.34) | <0.001* |
|  |  | Control | 3,248/16,839 (19.3) | | 1 |  | 1 |  |
| Smoking | | |  |  |  |  |  |  |
|  | Nonsmoker (n = 42,696) | |  | |  |  |  |  |
|  |  | Asthma | 1,581/6,808 (23.2) | | 1.25 (1.17-1.33) | <0.001* | 1.21 (1.14-1.29) | <0.001* |
|  |  | Control | 7,060/35,888 (19.7) | | 1 |  | 1 |  |
|  | Past smoker and current smoker (n = 15,239) | |  |  |  |  |  |  |
|  |  | Asthma | 447/1,830 (24.4) | | 1.43 (1.27-1.61) | <0.001* | 1.32 (1.17-1.50) | <0.001* |
|  |  | Control | 2,499/13,409 (18.6) | | 1 |  | 1 |  |
| Alcohol consumption | | |  |  |  |  |  |  |
|  | < 1 time a week (n = 41,375) | |  |  |  |  |  |  |
|  |  | Asthma | 1,564/6,570 (23.8) | | 1.29 (1.21-1.37) | <0.001* | 1.25 (1.16-1.33) | <0.001* |
|  |  | Control | 6,858/34,805 (19.7) | | 1 |  | 1 |  |
|  | ≥ 1 time a week (n = 16,560) | |  |  |  |  |  |  |
|  |  | Asthma | 464/2,068 (22.4) | | 1.27 (1.13-1.42) | <0.001* | 1.20 (1.06-1.35) | 0.003* |
|  |  | Control | 2,701/14,492 (18.6) | | 1 |  | 1 |  |
| Total cholesterol (mg/dL) | | |  |  |  |  |  |  |
|  | < 200 (n = 30,536) | |  |  |  |  |  |  |
|  |  | Asthma | 1,041/4,534 (23.0) | | 1.23 (1.14-1.33) | <0.001* | 1.19 (1.09-1.29) | <0.001* |
|  |  | Control | 5,127/26,002 (19.7) | | 1 |  | 1 |  |
|  | ≥ 200 to < 240 (n = 19,365) | |  |  |  |  |  |  |
|  |  | Asthma | 679/2,862 (23.7) | | 1.33 (1.21-1.46) | <0.001* | 1.30 (1.17-1.43) | <0.001* |
|  |  | Control | 3,158/16,503 (19.1) | | 1 |  | 1 |  |
|  | ≥ 240 (n = 8,034) | |  |  |  |  |  |  |
|  |  | Asthma | 308/1,242 (24.8) | | 1.41 (1.22-1.63) | <0.001* | 1.28 (1.10-1.49) | 0.001* |
|  |  | Control | 1,274/6,792 (18.8) | | 1 |  | 1 |  |
| Blood pressure (mmHg) | | |  |  |  |  |  |  |
|  | SBP < 140 and DBP < 90 (n = 42,113) | |  |  |  |  |  |  |
|  |  | Asthma | 1,474/6,270 (23.5) | | 1.25 (1.18-1.34) | <0.001* | 1.21 (1.13-1.30) | <0.001* |
|  |  | Control | 7,072/35,843 (19.7) | | 1 |  | 1 |  |
|  | SBP ≥ 140 or DBP ≥ 90 (n = 15,822) | |  |  |  |  |  |  |
|  |  | Asthma | 554/2,368 (23.4) | | 1.37 (1.23-1.52) | <0.001* | 1.30 (1.16-1.45) | <0.001* |
|  |  | Control | 2,487/13,454 (18.5) | | 1 |  | 1 |  |
| Fasting blood glucose (mg/dL) | | |  |  |  |  |  |  |
|  | < 100 (n = 37,254) | |  |  |  |  |  |  |
|  |  | Asthma | 1,262/5,415 (23.3) | | 1.25 (1.17-1.34) | <0.001* | 1.21 (1.12-1.30) | <0.001* |
|  |  | Control | 6,238/31,839 (19.6) | | 1 |  | 1 |  |
|  | ≥ 100 (n = 20,681) | |  |  |  |  |  |  |
|  |  | Asthma | 766/3,223 (23.8) | | 1.35 (1.23-1.48) | <0.001* | 1.28 (1.16-1.41) | <0.001* |
|  |  | Control | 3,321/17,458 (19.0) | | 1 |  | 1 |  |
| CCI score (score) | | |  |  |  |  |  |  |
|  | 0 (n = 39,123) | |  |  |  |  |  |  |
|  |  | Asthma | 1,142/4,914 (23.2) | | 1.33 (1.23-1.43) | <0.001* | 1.30 (1.21-1.40) | <0.001* |
|  |  | Control | 6,385/34,209 (18.7) | | 1 |  | 1 |  |
|  | 1 (n = 8,464) | |  |  |  |  |  |  |
|  |  | Asthma | 399/1,686 (23.7) | | 1.12 (0.98-1.27) | 0.093 | 1.00 (0.87-1.15) | 0.969 |
|  |  | Control | 1,509/6,778 (22.3) | |  |  | 1 |  |
|  | ≥ 2 (n = 10,348) | |  |  |  |  |  |  |
|  |  | Asthma | 487/2,038 (23.9) | | 1.29 (1.14-1.45) | <0.001* | 1.20 (1.06-1.36) | 0.005* |
|  |  | Control | 1,665/8,310 (20.0) | | 1 |  | 1 |  |

Abbreviations: CCI, Charlson comorbidity index; COM, chronic otitis media; COPD, chronic obstructive pulmonary disease; DBP, diastolic blood pressure; SBP, systolic blood pressure

* Logistic regression, Significance at P < 0.05

† A model 1 was adjusted for age, sex, income, and region of residence.

‡ A model 2 was adjusted for age, sex, income, region of residence, obesity, smoking, alcohol consumption, CCI scores, total cholesterol, SBP, DBP, fasting blood glucose, and COPD
