# Supplementary material for: Association between asthma or chronic obstructive pulmonary disease and chronic otitis media
Source: Sci Rep. 2022 Mar 10;12:4228. doi: 10.1038/s41598-022-08287-w (PMC8913729; doi:10.1038/s41598-022-08287-w)
Supplement: Supplementary file 2 — Supplementary Table S2. [file 41598_2022_8287_MOESM2_ESM.docx]

**S2 Table** Subgroup analyses of odds ratios (95% confidence interval) of COPD for COM according to obesity, smoking, alcohol consumption, total cholesterol, blood pressure, blood glucose, and CCI score

| Characteristics | | | No. of COM/ No. of participants (%) | | Odds ratios for COM | | | |
| --- | --- | --- | --- | --- | --- | --- | --- | --- |
|  |  |  |  |  | Model 1† | P-value | Model 2‡ | P-value |
| Obesity | | | | | | | | |
|  | Underweight (n = 1,435) | | | | | | | |
|  |  | COPD | 34/166 (20.5) | | 1.12 (0.74-1.71) | 0.589 | 0.87 (0.54-1.38) | 0.550 |
|  |  | Control | 221/1,269 (17.4) | | 1 |  | 1 |  |
|  | Normal weight (n = 20,389) | | | | | | | |
|  |  | COPD | 288/1,144 (25.2) | | 1.41 (1.22-1.62) | <0.001* | 1.28 (1.10-1.49) | 0.001 |
|  |  | Control | 3,761/19,245 (19.5) | | 1 |  | 1 |  |
|  | Overweight (n = 15,861) | | | | | | | |
|  |  | COPD | 179/747 (24.0) | | 1.24 (1.04-1.48) | 0.015 | 1.12 (0.93-1.34) | 0.244 |
|  |  | Control | 3,065/15,114 (20.3) | | 1 |  | 1 |  |
|  | Obese (n = 20,250) | | | | | | | |
|  |  | COPD | 262/1,023 (25.6) | | 1.43 (1.23-1.66) | <0.001* | 1.29 (1.11-1.51) | 0.001 |
|  |  | Control | 3,777/19,227 (19.6) | | 1 |  | 1 |  |
| Smoking | | |  |  |  |  |  |  |
|  | Nonsmoker (n = 42,696) | |  | |  |  |  |  |
|  |  | COPD | 524/2,139 (24.5) | | 1.31 (1.18-1.45) | <0.001* | 1.19 (1.07-1.33) | 0.001 |
|  |  | Control | 8,117/40,557 (20.0) | | 1 |  | 1 |  |
|  | Past smoker and current smoker (n = 15,239) | |  |  |  |  |  |  |
|  |  | COPD | 239/941 (25.4) | | 1.49 (1.27-1.74) | <0.001* | 1.31 (1.10-1.55) | 0.002 |
|  |  | Control | 2,707/14,298 (18.9) | | 1 |  | 1 |  |
| Alcohol consumption | | |  |  |  |  |  |  |
|  | < 1 time a week (n = 41,375) | |  |  |  |  |  |  |
|  |  | COPD | 578/2,322 (24.9) | | 1.32 (1.20-1.46) | <0.001* | 1.19 (1.08-1.33) | 0.001 |
|  |  | Control | 7,844/39,053 (20.1) | | 1 |  | 1 |  |
|  | ≥ 1 time a week (n = 16,560) | |  |  |  |  |  |  |
|  |  | COPD | 185/758 (24.4) | | 1.43 (1.20-1.70) | <0.001* | 1.33 (1.11-1.60) | 0.002 |
|  |  | Control | 2,980/15,802 (18.9) | | 1 |  | 1 |  |
| Total cholesterol (mg/dL) | | |  |  |  |  |  |  |
|  | < 200 (n = 30,536) | |  |  |  |  |  |  |
|  |  | COPD | 407/1,708 (23.8) | | 1.28 (1.14-1.44) | <0.001* | 1.17 (1.04-1.33) | 0.011 |
|  |  | Control | 5,761/28,828 (20.0) | | 1 |  | 1 |  |
|  | ≥ 200 to < 240 (n = 19,365) | |  |  |  |  |  |  |
|  |  | COPD | 227/965 (23.5) | | 1.28 (1.10-1.50) | 0.002 | 1.15 (0.97-1.35) | 0.100 |
|  |  | Control | 3,610/18,400 (19.6) | | 1 |  | 1 |  |
|  | ≥ 240 (n = 8,034) | |  |  |  |  |  |  |
|  |  | COPD | 129/407 (31.7) | | 1.92 (1.54-2.40) | <0.001* | 1.70 (1.35-2.14) | <0.001* |
|  |  | Control | 1,453/7,627 (19.1) | | 1 |  | 1 |  |
| Blood pressure (mmHg) | | |  |  |  |  |  |  |
|  | SBP < 140 and DBP < 90 (n = 42,113) | |  |  |  |  |  |  |
|  |  | COPD | 541/2,171 (24.9) | | 1.32 (1.19-1.47) | <0.001* | 1.21 (1.09-1.35) | 0.001 |
|  |  | Control | 8,005/39,942 (20.0) | | 1 |  | 1 |  |
|  | SBP ≥ 140 or DBP ≥ 90 (n = 15,822) | |  |  |  |  |  |  |
|  |  | COPD | 222/909 (24.4) | | 1.43 (1.22-1.68) | <0.001* | 1.27 (1.07-1.51) | 0.006 |
|  |  | Control | 2,819/14,913 (18.9) | | 1 |  | 1 |  |
| Fasting blood glucose (mg/dL) | | |  |  |  |  |  |  |
|  | < 100 (n = 37,254) | |  |  |  |  |  |  |
|  |  | COPD | 468/1,902 (24.6) | | 1.32 (1.18-1.47) | <0.001* | 1.20 (1.07-1.35) | 0.002 |
|  |  | Control | 7,032/35,352 (19.9) | | 1 |  | 1 |  |
|  | ≥ 100 (n = 20,681) | |  |  |  |  |  |  |
|  |  | COPD | 295/1,178 (25.0) | | 1.41 (1.23-1.63) | <0.001* | 1.27 (1.09-1.46) | 0.002 |
|  |  | Control | 3,792/19,503 (19.4) | | 1 |  | 1 |  |
| CCI score (score) | | |  |  |  |  |  |  |
|  | 0 (n = 39,123) | |  |  |  |  |  |  |
|  |  | COPD | 298/1,272 (23.4) | | 1.30 (1.14-1.49) | <0.001* | 1.17 (1.02-1.35) | 0.022 |
|  |  | Control | 7,229/37,851 (19.1) | | 1 |  | 1 |  |
|  | 1 (n = 8,464) | |  |  |  |  |  |  |
|  |  | COPD | 201/748 (26.9) | | 1.36 (1.15-1.62) | 0.001 | 1.30 (1.07-1.57) | 0.008 |
|  |  | Control | 1,707/7,716 (22.1) | | 1 |  | 1 |  |
|  | ≥ 2 (n = 10,348) | |  |  |  |  |  |  |
|  |  | COPD | 264/1,060 (24.9) | | 1.35 (1.16-1.58) | <0.001* | 1.23 (1.04-1.45) | 0.016 |
|  |  | Control | 1,888/9,288 (20.3) | | 1 |  | 1 |  |

Abbreviations: CCI, Charlson comorbidity index; COM, chronic otitis media; COPD, chronic obstructive pulmonary disease; DBP, diastolic blood pressure; SBP, systolic blood pressure

* Logistic regression, Significance at P < 0.05

† A model 1 was adjusted for age, sex, income, and region of residence.

‡ A model 2 was adjusted for age, sex, income, region of residence, obesity, smoking, alcohol consumption, CCI scores, total cholesterol, SBP, DBP, fasting blood glucose, and asthma
